# Evaluation of immature granulocyte parameters in myeloid neoplasms assayed by Sysmex XN hematology analyzer

**DOI:** 10.1007/s12308-022-00484-w

**Published:** 2022-02-08

**Authors:** Qifeng Lu, Ying Li, Tian Li, Tingting Hou, Yajuan Zhao, Shu Feng, Xixian Yang, Mengyu Zhu, Yajuan Shen

**Affiliations:** 1 Department of Clinical Laboratory, Shandong Provincial Hospital Affiliated to Shandong First Medical University, Jinan, 250021 Shandong Province China; 2 Department of Hematology, Shandong Provincial Hospital Affiliated to Shandong First Medical University, Jinan, 250021 Shandong Province China

**Keywords:** Immature granulocytes, Myeloid neoplasms, Hematology analyzer, Morphological abnormalities

## Abstract

Immature granulocytes (IGs) have significance for the diagnosis of myeloid neoplasms (MNs). The current study aims to use a hematology analyzer to evaluate the accuracy of IG parameters in MNs. Blood specimens from 388 patients with MN, 524 with non-hematological neoplasms (non-HNs), including 109 patients with inflammation and 68 undergoing G-CSF administration, and 500 healthy control subjects were analyzed. IG parameters was assayed by Sysmex XN-9000 (XN) and compared with manual assessments. A high level of agreement between IG% derived from XN and manual measurements for MN patients (*r* = 0.828, *p* < 0.0001) was revealed but only a moderate correlation for acute myeloid leukemia patients (AML; *r* = 0.597; *p* < 0.0001). Bland–Altman bias analysis was conducted, and the results showed that differences in IG% from XN and manual analysis for MN patients were considered clinically insignificant. ROC analysis demonstrated a good performance of IG# (AUC = 0.842) and IG% (AUC = 0.885) assessed by XN for MN patients with cut-off values of 0.200 × 10^9^/L and 1.95%, respectively. IG parameters from Sysmex XN analyzer are helpful for screening of MNs even though granulocyte morphological abnormalities may interfere with IG parameter accuracy.

**Supplementary Information:**

The online version contains supplementary material available at 10.1007/s12308-022-00484-w.

## Introduction

Immature granulocytes (IGs) comprise promyelocytes, myelocytes, and metamyelocytes [1]. Such cells are seldom seen in the peripheral blood (PB) of healthy subjects, except in certain physiological states and conditions, such as pregnancy or the neonatal period [2–4]. By contrast, IGs are found in patients’ PB in the following pathological conditions: acute infection, inflammation, tissue injury, intravascular hemolysis, acute hemorrhage, non-hematopoietic malignant tumor, hematological neoplasms and in those receiving granulocyte colony-stimulating factor (G-CSF) or granulocyte–macrophage colony-stimulating factor (GM-CSF) [5–13]. For patients undergoing G-CSF or GM-CSF treatment, IGs act as sensitive indices to indicate granulocytic hematopoietic recovery [12, 13]. IGs are also seen in patients with myeloid neoplasms (MNs), such as acute myeloid leukemia (AML), myeloproliferative neoplasms (MPN), myelodysplastic syndrome (MDS), and myelodysplastic/myeloproliferative neoplasms (MDS/MPN), and their presence can be used for diagnosis. The conditions of chronic myeloid leukemia (CML) and primary myelofibrosis (PMF) are particularly associated with an increase in IG level [11].

Prior to the availability of the automated hematology analyzer, Sysmex XN series, other analyzers, including Sysmex XS, XT, and XE series, were used to sort cells for research purposes [6, 14]. The Sysmex XN analyzer uses one of the six white blood cell (WBC) differential parameters in the WBC differential (WDF) channel to sort IGs. Erythrocytes are lysed by lysercell WDF which permeates the leukocyte membrane to allow fluorescent staining of nucleic acids by fluorocell™ WDF. A semiconductor laser is then used to detect cells by flow cytometry. A combination of side scatter light (SSC), reflecting complexity of intracellular structures, side fluorescence light (SFL), reflecting fluorescence intensity, and forward scatter light (FSC), to measure cell size, is combined by an intelligent gating algorithm provided by 00-19E (Build 2) IPU version software to judge WBC type. The WDF scattergram shows IGs as dark blue dots above neutrophils. The Q-flag represents the healthy maximal limit of IGs and, above this threshold, “IG present” is indicated. Previous studies have reported general evaluation of Sysmex XN performance in measuring IG parameters [15–17], but application of this approach to MNs has been neglected.

The current study aimed to investigate Sysmex XN series performance in IG quantification in various MNs by comparison with manual measurements. Evaluation of the suitability of the Sysmex XN analyzer in reliable detection of IGs in PB and screening for MNs was assessed.

## Materials and methods

### Blood samples

A total of 1412 blood samples, including 388 from MN patients, 524 from patients with non-hematological neoplasms (non-HNs), and 500 from healthy control subjects, taken between November 2016 and December 2019 in Shandong Provincial Hospital Affiliated to Shandong First Medical University, Jinan, China, were included in the study.

MN patients were enrolled from the Department of Hematology and diagnosed according to 2016 WHO classification criteria [11, 18]. Specimens were collected at first diagnosis prior to chemotherapy or recurrence. Data from each individual was included in the study only once and MN patients without complete data were excluded. Characteristics of all MN patients are shown in Table 1. Non-HN patients were enrolled from departments other than hematology, including 284 from internal medicine, 177 from surgery, and 63 from ophthalmology, otolaryngology, stomatology, gynecology, neurology, dermatology, or traditional Chinese medicine. Non-HN patients included 109 with inflammation from respiratory medicine, intensive care unit, and rheumatology and surgery department, and 68 undergoing recombinant human G-CSF administration from the oncology department. Healthy individuals were enrolled from the physical examination center with the exclusion of pregnant women.

**Table 1:** Characteristics of MN patients

Diagnosis	Cases
AML and related precursor neoplasms	231
AML with recurrent genetic abnormalities	83
AML with RUNX1-RUNX1T1	7
AML with CBFB-MYH11	10
Acute promyelocytic leukemia with PML-RARA	42
AML with mutated NPM1	10
AML with biallelic mutation of CEBPA	10
AML with mutated RUNX1	4
AML with myelodysplasia-related changes	5
Therapy-related AML	3
AML, NOS	140
AML without maturation	6
AML with maturation	52
Acute myelomonocytic leukemia	41
Acute monoblastic and monocytic leukemia	41
Myeloproliferative neoplasms	77
CML	47
Primary myelofibrosis	30
MDS	63
Myelodysplastic/myeloproliferative neoplasm	17
Chronic myelomonocytic leukemia	10
Myelodysplastic/myeloproliferative neoplasm, unclassifiable	7
Total MN	388

Anticoagulant PB samples were taken with EDTA (Becton Dickinson, San Jose, USA) for routine blood analysis. The study was approved by the medical Ethics Committee of Shandong Provincial Hospital (No.2016-KY-051).

### Sample analysis

The fully automated Sysmex XN series employs the automated hematology analyzer, XN-9000 (Sysmex, Kobe, Japan), equipped with 00-19E (Build 2) IPU version and the automated slide making/staining device, Sysmex SP-10 (Sysmex, Kobe, Japan). Annual calibration of the XN analyzer was performed, and precision, linearity, carryover, and stability were evaluated regularly. Measurement uncertainty, the dispersion of the measured value reasonably assigned by the characterization, was 0.03 × 10^9^/L for IG# from XN, 0.2% for IG% from XN, and 0.8% for IG% from manual methods. Two levels of blood XN check™ were conducted daily to monitor equipment performance and incorporated into the Sysmex program for real-time quality control. All MN and non-HN PB samples were initially analyzed by XN-9000; blood smears were made and stained using SP-10 and Wright Giemsa (Muto Pure Chemicals, Tokyo, Japan). Control samples were assayed by XN-9000, only, and no blood smears made. All samples were analyzed within 4 h of collection. All threshold values of Q-flags (“IG present”, “Left shift”, “Blast”, “Abn lympho”, and “Atypical lympho”) were set at 100. The WBC#, RBC#, HGB#, PLT#, IG#, IG%, instrument flags, and WDF scattergrams of all samples were recorded.

Two experienced technologists manually classified 200 cells per smear using an Olympus BX53 microscope according to the Clinical and Laboratory Standards Institute (CLSI) H20-A2 [19]. The mean IG%, composed of promyelocytes, myelocytes, and metamyelocytes, calculated by the 2 technologists was considered to be the final result. In the event of significant differences between the results from the 2 technologists, a third technologist classified the slide.

### Statistical analysis

Non-parametrical tests (Mann–Whitney test) were performed to compare IG parameters between 2 different groups and the Kruskal–Wallis test to compare 3 or more groups. Spearman rank correlation coefficient analysis was used to evaluate the relationship between IG% values obtained from the Sysmex XN analyzer and from manual assessment. Values for *r* coefficients < 0.30 were considered to present negligible correlations; 0.30–0.50 low correlations; 0.50–0.70 moderate correlations; 0.70–0.90 strong correlations; and ≥ 0.90 a very high correlation [20]. Bland–Altman bias analysis was performed to compare IG% differences from the two methods. Receiver operating characteristic (ROC) curve analysis was conducted to estimate sensitivity, specificity, and cut-off value for IG parameters assayed by XN analyzer. Pairwise comparisons of the area under the ROC curves (AUCs) were performed using Z-test.

MedCalc Software 19.1 (Ostend, Belgium) and IBM SPSS Statistics 18.0.0 (Chicago, USA) were used. A value of *p* < 0.05 was considered to be statistically significant.

## Results

### Comparison of XN-derived IG# and IG% values and manually-derived IG% in PB from MN, non-HN and control subjects

**Fig. 1:**
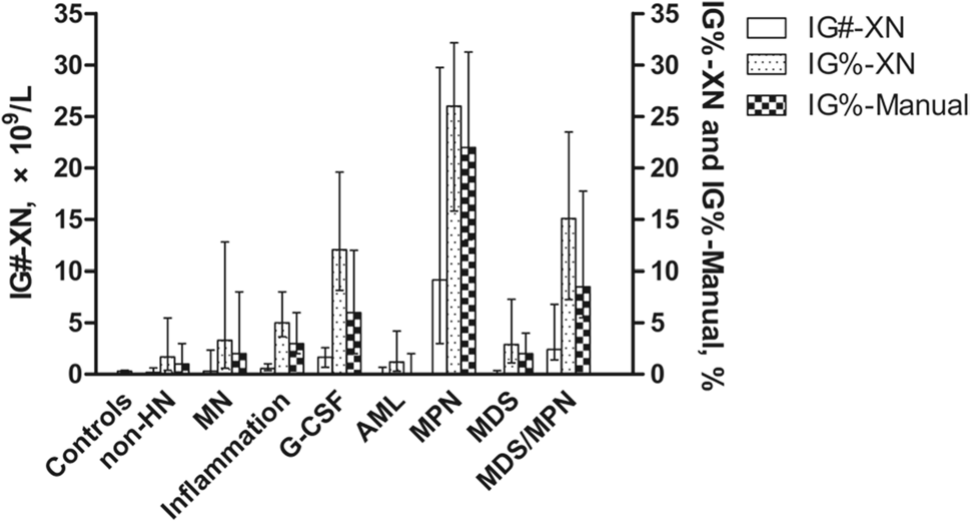
Comparison of IG#-XN, IG%-XN and IG%-manual values across groups. Columns and bars represent median with interquartile range

Increased IG# (both *p* < 0.0001) and IG% (both *p* < 0.0001) from XN series measurements were observed in MN and non-HN patients when compared with healthy controls (Fig. 1). IG% in healthy controls was not analyzed manually as the positive ratios were too low. Subgroups of patients with inflammation and receiving G-CSF showed higher levels of IG# and IG% from XN analysis plus IG% from manual measurements than the entire group of non-HN patients (all *p* < 0.0001; Fig. 1). Subgroups of MN patients with MPN (CML and PMF) showed the highest levels of XN-derived IG# and IG% values and also IG% from manual measurements (all *p* < 0.0001) when compared with other subgroups (Fig. 1).

### Correlations and differences between XN and manual estimates of IG% in MN and non-HN patients

Strong correlations between XN- and manually derived IG% values were found in both MN and non-HN patients (Table 2). Although correlations were statistically significant in all groups of patients with AML, MPN, MDS, and MDS/MPN, only moderate correlations were shown for AML patients (Table 2).

**Table 2:** Spearman rank correlation analysis of XN- and manually derived IG% values in PB from MN and non-HN patients

	*N*	Correlation coefficient ( r)	*p* value
Non-HN	524	0.861	< 0.0001
Inflammation	109	0.717	< 0.0001
G-CSF	68	0.762	< 0.0001
MN	388	0.828	< 0.0001
AML	231	0.597	< 0.0001
MPN	77	0.873	< 0.0001
MDS	63	0.767	< 0.0001
MDS/MPN	17	0.750	0.0005

**Fig. 2:**
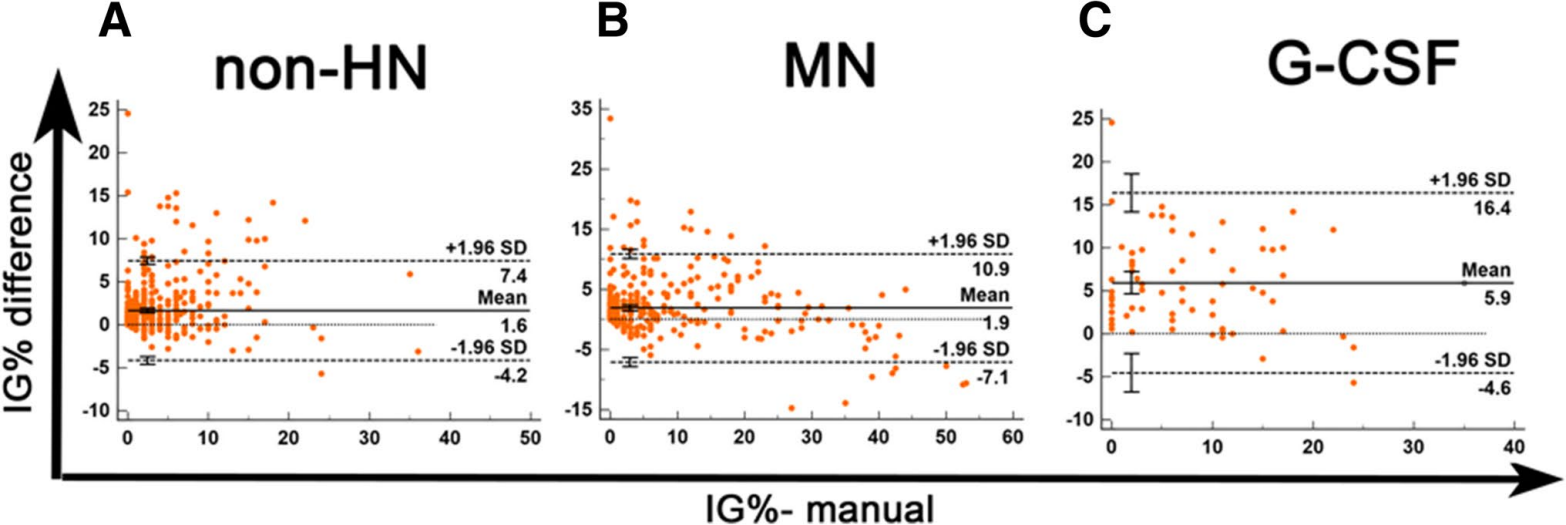
Comparison of XN- and manually-derived IG% values using Bland–Altman plot. The difference between two values (XN IG% – manual IG%) was plotted on the y-axis against manual IG% on the x-axis. Solid lines: mean differences; dashed lines: mean difference ± 1.96 SD

Differences between XN- and manually derived IG% values were compared using Bland–Altman bias analysis, and differences for both MN and non-HN patients were considered clinically insignificant (Fig. 2). However, a relatively high difference in the mean and in the 95% CI of limits of agreement (LoA) was shown in the G-CSF subgroup (Fig. 2).

### Analysis of ROC curve and cut-off values of XN IG# and IG% from MN and non-HN patients

ROC analysis was performed to assess XN-derived values of IG# and IG% in detection of IGs in PB. IG% values from manual inspection were regarded as the gold standard against which the IG# and IG% values from XN measurements were compared. The AUCs of XN-derived IG# and IG% values were above 0.8 for both MN and non-HN patients, indicating a good level of detection (Table 3). Comparison of the AUCs for XN-derived IG# and IG% values for MN patients revealed that the IG% values showed better efficiency than IG# (*p* < 0.05). Both the XN-derived IG# and IG% parameters showed higher efficiency in non-HN than in MN patients (*p* < 0.05). The Youden index gave cut-off values of 0.200 × 10^9^/L for IG# and 1.95% for IG% (Table 3).

**Table 3:** ROC curve and cut-off values for XN-derived IG# and IG% values in PB from MN and non-HN patients

	N	AUC	SE	95% CI^a^	*p* value	Cut-off	Sensitivity	Specificity	Youden index
IG#-XN, × 10^9^/L						0.200			
Non-HN	524	0.918	0.013	0.892 to 0.943	< 0.0001		0.767	0.886	0.653
MN	388	0.842	0.002	0.803 to 0.881	< 0.0001		0.746	0.791	0.537
IG%-XN, %						1.95			
Non-HN	524	0.937	0.011	0.916 to 0.958	< 0.0001		0.787	0.934	0.721
MN	388	0.885	0.017	0.851 to 0.918	< 0.0001		0.813	0.784	0.597

## Discussion

IGs are often found in the PB of patients with acute infection, inflammation, acute hemorrhage, and those receiving G-CSF. The non-HN patients enrolled in the current study included a number of patients with these conditions accounting for the finding that IG levels among non-HN patients were higher than those of controls.

IGs are easily distinguishable from mature leukocytes in the WDF channel due to their larger nuclear volume and higher fluorescent intensity. This observation underlies the strong correlations between IG% values measured by XN and those from manual inspection in non-HN and MN PB samples. However, morphological abnormalities of immature and mature granulocytes confuse the identification of IGs, resulting in inaccurate counts.

Overall comparisons of WDF scattergrams revealed that inconsistencies between IG% values from XN and manual measurements were usually due to inaccurate IG gating in the scattergram (Supplemental Fig. S1). Granulocytes with morphological abnormalities could be identified in blood smears from all of the inconsistent cases (Supplemental Fig. S2). This phenomenon was observed among samples from patients with acute promyelocytic leukemia (APL), acute myelomonocytic leukemia (AML-M4), and acute monoblastic and monocytic leukemia (AML-M5). The 2015 International Council for Standardization in Haematology (ICSH) recommended that abnormal promyelocytes in APL samples should be counted as blast equivalents rather than as IGs [1]. In addition, neutrophil dysplasia, such as hypogranulation and hyposegmentation, may also be present in AML samples [1, 11] and might interfere with IG gating, leading to inconsistencies. Moreover, “left shift”, arising from elevation of percentages of band neutrophils (> 5%) and cells with hypergranulation, vacuolation, and cytosolic Dӧhle bodies [1, 21], often follows G-CSF administration and may interfere with the intelligent software system gating and falsely raise IG count.

In summary, the IG parameters derived from Sysmex XN hematology analyzer efficiently indicate the presence of IG in PB of MN patients, and this approach may facilitate screening for MNs. In addition, inaccuracies in XN-derived IG parameter measurements may arise due to the presence of granulocytes with morphological abnormalities in PB samples.

### Supplementary Information

Below is the link to the electronic supplementary material.

Supplemental Figure S1: InaccurateIG gating in APL, AML-M4 and G-CSF administration WDF scattergrams. Dark bluedots in circles are identified as IGs by Sysmex XN. APL: acute promyelocytic leukemia;AML-M4: acute myelomonocytic leukemia. (JPG 229 KB)

SupplementalFigure S2: Morphological granulocyte abnormalities in PB from APL, AML-M4 andG-CSF treated patients. Blood smears are magnified x1000 and visualized with WrightGiemsa stain. Red arrow points: abnormal promyelocytes; white arrow points: hypogranulatedneutrophils; blue arrow points: hypergranulated neutrophils. (JPG 180 KB)

## Data Availability

Available on request.
