# Supplementary figures and images for: Evaluation of immature granulocyte parameters in myeloid neoplasms assayed by Sysmex XN hematology analyzer
Source: J Hematop. 2022 Feb 8;15(1):1–6. doi: 10.1007/s12308-022-00484-w (PMC10869398; doi:10.1007/s12308-022-00484-w)

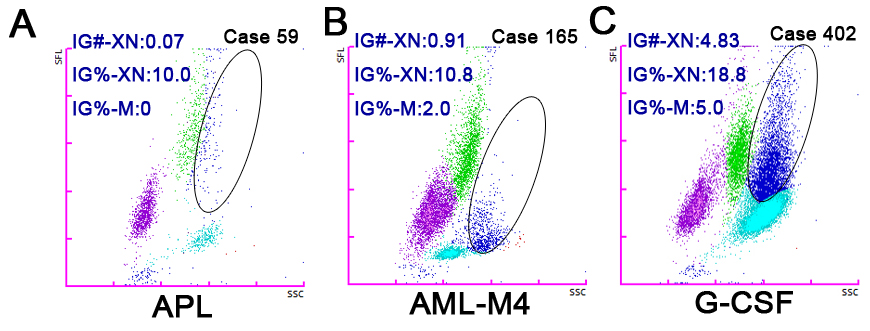

Supplement: Supplementary file 1 — Supplemental Figure S1: InaccurateIG gating in APL, AML-M4 and G-CSF administration WDF scattergrams. Dark bluedots in circles are identified as IGs by Sysmex XN. APL: acute promyelocytic leukemia;AML-M4: acute myelomonocytic leukemia. (JPG 229 KB) [file 12308_2022_484_MOESM1_ESM.jpg]

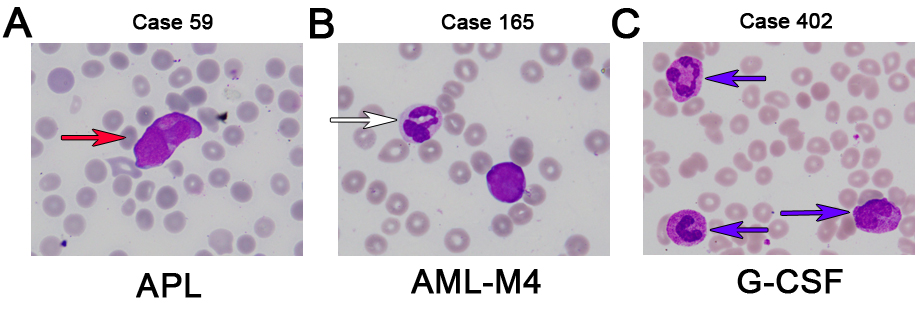

Supplement: Supplementary file 2 — SupplementalFigure S2: Morphological granulocyte abnormalities in PB from APL, AML-M4 andG-CSF treated patients. Blood smears are magnified x1000 and visualized with WrightGiemsa stain. Red arrow points: abnormal promyelocytes; white arrow points: hypogranulatedneutrophils; blue arrow points: hypergranulated neutrophils. (JPG 180 KB) [file 12308_2022_484_MOESM2_ESM.jpg]
